# Diagnostic accuracy of non-invasive tools to detect esophageal varices keeping esophagogastroduodenoscopy as gold standard in patients with compensated chronic liver disease

**DOI:** 10.12669/pjms.42.9.16288

**Published:** 2026-09

**Authors:** Nayab Munib, Iqbal Haider, Mahnoor Khattak

**Affiliations:** 1 Nayab Munib, Department of Medicine, Khyber Teaching Hospital, Peshawar, Pakistan; 2 Iqbal Haider, Department of Medicine, Medical Teaching Institution, Khyber Teaching Hospital, Peshawar Pakistan; 3 Mahnoor Khattak, Department of Medicine, Khyber Teaching Hospital, Peshawar, Pakistan

**Keywords:** Aspartate Platelet Count, Aminotransferase, Chronic Liver Disease, Esophageal varices, Endoscopy, Liver Cirrhosis, Portal Hypertension

## Abstract

**Objectives:**

To determine the diagnostic accuracy of Aspartate Aminotransferase to Platelet Ratio Index (APRI) and Platelet Count to Spleen Diameter Ratio (PC/SD) having upper esophagogastroduodenoscopy findings as the reference standard for predicting esophageal varices in patients with compensated chronic liver disease.

**Methodology:**

This prospective cross-sectional validation study was carried out at the Department of Medicine, Khyber Teaching Hospital (KTH) Peshawar from August 2025 to January 2026. A total of 161 consecutive patients aged 20 to 70 years with compensated chronic liver disease (CLD) who fulfilled the eligibility criteria were enrolled using non probability consecutive sampling technique. Patients with peptic ulcer disease, contraindications to upper gastrointestinal endoscopy, hepatocellular carcinoma or altered upper gastrointestinal anatomy were excluded. APRI and PC/SD ratio were calculated for all participants and upper gastrointestinal endoscopy was performed as the reference standard. Diagnostic performance was evaluated by calculating sensitivity, specificity, positive predictive value, negative predictive value and overall diagnostic accuracy.

**Results:**

A total of 161 patients with CLD were included, of whom 101 (62.7%) had esophageal varices (EVs) on upper gastrointestinal endoscopy. The APRI score demonstrated 100% sensitivity, 65.0% specificity and an overall diagnostic accuracy of 87.0%, whereas the PC/SD ratio showed 51.5% sensitivity, 100% specificity, and 69.6% accuracy. Both markers demonstrated excellent discriminatory performance, with AUCs of 0.943 for APRI and 0.939 for the PC/SD ratio (p = 0.315). APRI was significantly more sensitive, while the PC/SD ratio was significantly more specific for detecting EVs (both p < 0.001). Increasing APRI values and decreasing PC/SD ratio values were significantly associated with higher grades of EVs (p < 0.001).

**Conclusion:**

Both the APRI and PC/SD ratio demonstrated acceptable diagnostic performance for identifying EVs in patients with CLD. These inexpensive and readily available biomarkers may assist in risk stratification and prioritizing patients for endoscopic evaluation particularly in resource limited settings. However, upper gastrointestinal endoscopy remains the reference standard for the diagnosis of EVs.

## INTRODUCTION

Acute variceal bleed is a fatal complication of chronic liver disease with 20.0% mortality rate. Primary prevention of variceal bleed reduces risk of bleeding and mortality.^1^ Primary prevention is achieved using pharmacological and endoscopic measures. Endoscopic measures include band ligation for high risk engorged esophageal varices and injection sclerotherapy for gastric varices.^2^

The approximate prevalence of esophageal and gastric varices in decompensated cirrhosis is 70.0%, however, 40.0% of compensated liver cirrhosis are found having varices on screening endoscopy.^3^ However, the expertise, instrumentation and cost are some of the major factors, limiting the application of endoscopy in resource limited settings.^4^ The on-going conquest to explore relatively non-invasive techniques for predicting engorged varices has drawn attention in the last few years. APRI (Aspartate transaminase to platelet count ratio index) and platelet count to spleen diameter ratio index are two such non-invasive tools.^5^

APRI score was first described for hepatic fibrosis secondary to hepatitis C virus. The predictive ability of APRI score is attributed to portal hypertension secondary to hepatic fibrosis and sequestration of platelets in enlarged spleen.^6^ Link between esophageal varices (EVs) and APRI score was reported in a study irrespective of the cause of hepatic fibrosis.^7^ Taking 0.5 threshold as cut off, sensitivity and specificity of APRI score in predicting EVs was 81.0% and 50.0% respectively in a study.^8^

Platelet count (PC) to spleen diameter (SD) ratio index was first described by Gianinni et al. It is convenient in predicting varices in cirrhotic patients with better diagnostic accuracy. In this study the sensitivity and specificity of PC to SD ratio was 98.1% and 88.6% for predicting varices in cirrhotic patients respectively.^9^

Although several international studies have evaluated APRI and the PC/SD ratio, evidence from Pakistan remains limited and inconsistent. Differences in the epidemiology of CLD, particularly the high prevalence of hepatitis B and hepatitis C virus infections, variations in healthcare infrastructure, and restricted access to endoscopic services may influence the diagnostic performance of these non-invasive markers. Therefore, local validation is necessary before these tools can be incorporated into routine clinical practice. APRI score and PC/SD ratio are seldom studied locally for predicting EVs in CLD patients in our settings. Hence the study has been planned to evaluate the diagnostic accuracy of both ratios for EVs. Results of this study would benefit clinicians in improving early diagnosis and risk stratification for variceal bleeding and other complications of portal hypertension in CLD patients.

## METHODOLOGY

This cross-sectional validation study was carried out at the Department of Medicine, Khyber Teaching Hospital, Peshawar from 01-08-2025 to 30-01-2026.

### Ethical Approval:

after taking approval from the research review board vide letter no# 547DME/KMC, dated; May 29, 2025.

A total of 161 male and female patients aged 20 to 70 years diagnosed with compensated CLD were studied for diagnostic accuracy of non-invasive APRI score and PC/SD ratio for predicting EVs taking endoscopy as gold standard. Compensated CLD was diagnosed based on clinical findings, laboratory investigations, and abdominal ultrasonography demonstrating coarse heterogeneous hepatic echotexture, surface nodularity, irregular liver margins, and reduced liver size, with no previous history of decompensating events including ascites, hepatic encephalopathy or EVs. The diagnostic criteria were based on current international recommendations for compensated advanced chronic liver disease. Patients with peptic ulcer disease, contraindication to upper GI endoscopy, hepatocellular carcinoma on ultrasound (U/S) were excluded while those presenting with compensated CLD were included in the study.

The equation, APRI = [Patients AST (IU/L) ÷ Upper normal limit of AST, i.e. 40 (IU/L)]/platelet count (10^9^/L) was used. APRI >0.5 was considered positive for EVs. Platelet count/spleen diameter was calculated using the equation PC/SD = Platelet count (cells/mm³)/Spleen diameter (mm). PC/SD ≤884 was considered positive for EVs. Direct observation of engorged protruded vessels from esophageal wall into the lumen under white light endoscopy was considered confirmatory for EVs. Continuous variables were tested for normality using the Shapiro–Wilk test and reported as mean ± standard deviation or median (interquartile range) as appropriate; categorical variables were reported as frequencies and percentages. The diagnostic accuracy of APRI (cut-off >0.5) and the platelet count/spleen diameter (PC/SD) ratio (cut-off ≤884, computed as platelet count in cells/mm³ divided by spleen bipolar diameter in millimetres) against upper gastrointestinal endoscopy as the reference standard was expressed as sensitivity, specificity, positive predictive value, negative predictive value, and overall accuracy, each with a Wilson score 95% confidence interval. Receiver operating characteristic (ROC) curve analysis was performed for both markers as continuous variables, with the area under the curve (AUC) reported with a DeLong 95% confidence interval; the two correlated AUCs were compared using the DeLong test. Youden’s index was used to identify the empirically optimal cut-off in this cohort for comparison with the literature-derived cut-offs. Because APRI and PC/SD were assessed in the same patients, their paired sensitivities and paired specificities were compared using McNemar’s test (exact binomial form where the number of discordant pairs was small). Categorical associations with variceal presence were assessed using the chi-square test, or Fisher’s exact test where any expected cell count was below five, and effect sizes are additionally reported as odds ratios with 95% confidence intervals. A two-sided p-value <0.05 was considered statistically significant. Analyses were performed in Python (SciPy 1.17, scikit-learn 1.8).”

## RESULTS

The descriptive statistics, demographics along with clinical characteristics of the study cohorts are reported in Table-I and Table-II. The study included 161 patients and the marked difference between the mean and median APRI and PC/SD values indicates a skewed distribution of these non-invasive markers within the study population.

**Table-I:** Descriptive statistics of participants

Parameter	Value
Age(years), mean ± SD	49.4±10.6
BMI (Kg/m²), mean ± SD	26.6±2.4
AST(U/L), mean ± SD	85.6±46.9
Platelet count (×10^9^/), mean± SD	167.5±54.0
Spleen diameter(cm), mean± SD	12.39±2.31
APRI score, mean± SD	1.73±1.51
APRI score, median (IQR)	0.77(0.52-2.99)
PC/SD ratio (cells/mm³ per mm), mean ±SD	1479.55±709.3
PC/SD ratio, median (IQR)	154.24(777.0-2028.33)

***Note:*** APRI is unit less. PC/SD is reported in cells/mm³ per mm, consistent with the ≤884 cut-off.

**Table-II:** Demographics and clinical Characteristics

Variable	Category	n (%)
Age Groups (in Years)	20-40	45(28%)
	41-60	78(48.4%)
	61-70	38(23.6%)
Gender	Male	82(50.9%)
	Female	79(49.1%)
Residence	Urban	81(50.3%)
	Rural	80(49.7%)
Education	No formal	28(17.4%)
	Primary	42(26.1%)
	Secondary	58(30.0%)
	Higher	33(20.5%)
Profession	Employed	89(55.3%)
	Unemployed	72(44.7%)
Socioeconomic status	Low	38(23.6%)
	Middle	86(53.4%)
	High	37(23.0%)
CLD etiology	Viral Hepatitis	89(55.3%)
	NAFLD (Non-Alcoholic Fatty Liver Disease)	32(19.9%)
	Autoimmune	24(14.9%)
	Other	16(9.9%)

Demographic and clinical characteristics analysis showed that 101 (62.7%) of the 161 patients had EVs on upper gastrointestinal endoscopy, while 60 (37.3%) had no varices. The prevalence of varices increased significantly with advancing age and was higher among male patients. Viral hepatitis was the most common etiology associated with EVs, whereas autoimmune liver disease was associated with lower odds of variceal presence. Overall, the distribution of patient characteristics demonstrated significant associations between several demographic and clinical variables and the presence of EVs is shows in Table-III. The agreement between the non-invasive markers and OGD findings for the detection of EVs is shown in Tabnle-III. APRI (>0.5) identified all patients with varices, achieving 100% sensitivity, although 21 patients without varices were falsely classified as positive. In contrast, the PC/SD ratio (≤884) showed 100% specificity with no false-positive results but missed 49 patients with varices, reflecting its lower sensitivity.

**Table-III:** Contingency Table Analysis – APRI and PC/SD ratio vs OGD (Gold Standard), (n = 161)

	OGD Positive (n = 101)	OGD Negative (n = 60)	Total (n = 161)
APRI Positive (>0.5)	101 (100%)	21(35.0%)	122
APRI Negative (<0.5)	0(0.0%)	39(65.0%)	39
PC/SD Positive (<884)	52(51.5%)	0(0.0%)	52
PC/SD Negative (>884)	49 (48.5%)	60 (100.0%)	109

The analysis of diagnostic performance of APRI and PC/SD ratio for predicting EVs using OGD as the reference demonstrated following results. APRI demonstrated 100% sensitivity, 65.0% specificity, and an overall diagnostic accuracy of 87.0% whereas the PC/SD ratio showed 51.5% sensitivity, 100% specificity and 69.6% accuracy. Both markers exhibited excellent discriminatory ability with AUCs of 0.943 for APRI and 0.939 for the PC/SD ratio, with no significant difference between them (p = 0.315). These findings indicate that APRI is more suitable as a screening tool, while the PC/SD ratio is more useful for confirming the presence of EVs (Table-IV).

**Table-IV:** Diagnostic Accuracy with 95%CI

Parameter	APRI (95%CI)	PC/SD (95% CI)
Sensitivity	100.0 %	51.5% (41.9-61.0)
Specificity	65.0%	100.0% (94.0-100.0)
Positive Predictive Value	82.8%	100.0% (93.1-100.0)
Negative Predictive Value	100.0%	55.0% (45.7-64.1)
Overall Accuracy	87.0%	69.6% (62.1-76.1)

95% CIs are Wilson score intervals. Paired comparison (McNemar, exact binomial form): sensitivity difference p<0.0001; specificity difference p<0.0001 — i.e. on this dataset APRI is significantly more sensitive and PC/SD significantly more specific.

ROC curve analysis showed excellent diagnostic performance for both markers, with an AUC of 0.943 for APRI and 0.939 for the PC/SD ratio. There was no significant difference between the two AUCs (DeLong test, p = 0.315), indicating comparable overall discriminative ability (Table-V).

**Table-V:** ROC/AUC Analysis

Marker	AUC	95%CI (DeLong)	Youden Optimal Cut-off (this cohort)	Literature cut-off used
APRI	0.943	0.910-0.976	>0.64	>0.5
PC/SD	0.939	0.905-0.973	<1684	<884

Analysis of performance of APRI and PC/SD ratio in predicting clinically significant (Grade II-III) EVs and their relationship with variceal severity. APRI showed 100% sensitivity but lower specificity 37.1%, whereas the PC/SD ratio demonstrated 92.9% sensitivity and 100% specificity. Both markers exhibited a significant association with increasing variceal grade (Kruskal–Wallis, p < 0.001). Median APRI values increased progressively, while median PC/SD values decreased with worsening variceal grades (Table-VI, VII and VIII).

**Table-VI:** High Risk Varices Analysis

Marker	Sensitivity for Grade >II (95% CI)	Specificity for Grade>ll (95%CI)
APRI	100.0 % (93.6-100.0)	37.1% (28.5-46.7)
PC/SD	92.9% (83.0-97.2)	100.0% (96.5-100.0)

**Table-VII:** Grade Severity

Grade	n	APRI (Median)	PC/SD, Median
No varices	60	0.46	2265
Grade I	45	0.73	1583
Grade II	31	2.99	777
Grade III	25	4.38	568

**Table-VIII:** Esophageal Varices Stratification with Baseline Parameters (n=161)

Variable	Comparison (Ref. Category)	Odds ratio (95%CI)	Overall p
Age Group	41-60 vs 20-40	3.10 (1.42-6.74)	<0.001 †
	61-70 vs 20-40	8.32 (2.62-26.39)	
Gender	Male vs female	4.21 (2.12-8.37)	<0.001
BMI Category	Overweight vs normal	2.28 (1.12-4.64)	0.043
	Obese vs normal	0.96 (0.29-3.15)	
Residence	Urban vs rural	0.56 (0.29-1.07)	0.076
Education	Higher vs No formal	0.10 (0.03-0.37)	<0.001 †
	Primary vs No formal	1.02 (0.40-2.59)	
	Secondary vs No formal	1.15 (0.47-2.83)	
Profession	Employed vs Unemployed	0.69 (0.36-1.32)	0.262
Socioeconomic status	High vs Low	0.20 (0.07-0.54)	<0.001 †
	Middle vs Low	1.48 (0.65-3.36)	
CLD etiology	Autoimmune vs NAFLD	0.25 (0.09-0.76)	<0.001 ‡
	Other vs NAFLD	0.02 (0.00-0.32)	
	Viral Hepatitis vs NAFLD	2.94 (1.18-7.31)	

† Chi-square, all expected cell counts ≥5. ‡ Fisher’s exact test used (the “Other” etiology cell has small/zero counts that violate chi-square assumptions). No correction for multiple comparisons was applied across the eight stratifying variables.

Table-IX presents the multivariable logistic regression analysis of factors associated with EVs. After adjustment for potential confounders, male gender and viral hepatitis etiology remained significant independent predictors of EVs, whereas age and BMI were not independently associated. These findings indicate that gender and the underlying etiology of CLD are stronger predictors of variceal presence than age or body mass index in this cohort.

**Table-IX:** Multivariable logistic regression

Predictor	Adjusted OR	95% CI
Age (per year)	1.02	0.89-1.14
BMI (per unit)	0.69	0.43-1.11
Gender (male vs female)	5.65	2.21-26.42
Etiology: NAFLD vs Autoimmune	6.07	1.82-38.39
Etiology: viral hepatitis vs Autoimmune	12.77	4.65-71.55
Etiology: Others vs Autoimmune	~0 (unstable)	Quasi-separation

## DISCUSSION

The current study examined the clinical and demographic parameters linked to variceal presence, as well as the diagnostic value of the APRI and PC/SD ratio to identify EVs among individuals with CLD. In line with the evolving pattern of CLD in nations with limited resources, our cohort’s demographic makeup showed a mean age in the fifth decade with a slight male predominance.^10^ Due to the persistent burden of hepatitis B and C infections in resource limited setting like Pakistan, viral hepatitis became the most common cause. Viral hepatitis continues to be the primary cause of cirrhosis globally, especially in Asia and Africa, according to latest estimates, which are consistent with this distribution.^11^ Despite being lower than in Western cultures, our cohort’s incidence of NAFLD highlights the growing trend of metabolic liver damage in developing nations going through dietary transition.^12^

This study had a 62.7% (101/61) general prevalence of EVs, which is within the commonly reported range among individuals with compensated cirrhosis.^13^ The prevalence of EVs in the present study was higher than that reported by Zoughlami et al., who found an overall prevalence of 44.8% among patients with compensated advanced CLD.^14^ Given the high incidence, there is a clinical need for efficient non-invasive screening methods, especially in environments with limited resource settings.

Our results show that APRI performs better in terms of diagnostic accuracy sensitivity 100% ; negative predictive value 100%) than the pooled estimates presented in a thorough meta-analysis by Shanka NY et al., who reported a pooled sensitivity of 97% for liver stiffness combined with platelet count in detecting high-risk EVs, although specificity was relatively low (41%).^15^ This disparity might be explained by variations in the research populations, namely the prevalence of viral hepatitis-related cirrhosis in our cohort, with which APRI was first created and validated. Sharaf et al. found that although APRI correlated with the presence of high risk EVs, its predictive performance was inferior to ALBI-PLT score which remained out of scope of this study. Consequently, APRI alone was considered less reliable for identifying patients with high-risk varices who require endoscopic screening.^16^

In our study, the PC/SD ratio showed similar sensitivity to APRI but somewhat greater specificity (specificity 100%; positive predictive value 100%). In the study conducted by Pastrovic et al. the authors evaluated several biochemical markers, including APRI and PC/SD ratio, for non-invasive prediction of high risk EVs in patients with compensated liver disease. The study found that APRI had only diagnostic value.^17^

An important finding of this study was the excellent overall discriminative ability of both markers. The ROC analysis demonstrated an AUC of 0.943 for APRI and 0.939 for the PC/SD ratio, with no statistically significant difference between the two markers (p = 0.315). This indicates that both indices possess comparable ability to distinguish patients with and without EVs when analyzed as continuous variables. The observed differences in sensitivity and specificity are therefore largely attributable to the fixed literature- derived cut-off values rather than to differences in their intrinsic diagnostic performance. This observation is clinically relevant because it suggests that locally optimized thresholds may improve diagnostic accuracy in different populations. Even greater diagnosis accuracy at a particular cut-off value was found by Suttitossatam N et al. in research involving cirrhotic patients.^18^ In a meta-analysis by Deng et al including 12 studies, APRI showed only moderate diagnostic accuracy, with a pooled AUROC of 0.677, pooled sensitivity of 60% and specificity of 67% for detecting EVs.^19^

When clinically significant (Grade II–III) varices were analyzed, APRI maintained 100% sensitivity, whereas the PC/SD ratio achieved 100% specificity. These findings have practical implications because highly sensitive tests are preferred for initial screening to minimize missed cases, whereas highly specific tests are valuable for confirming disease and potentially reducing unnecessary endoscopy. Nevertheless, because red wale signs were not recorded separately, Grade II–III varices were used as an operational surrogate for high-risk varices rather than the complete Baveno VII definition. This should be considered when interpreting these findings. Recent refinements of the Baveno VII recommendations continue to emphasize the role of validated non-invasive tools in reducing unnecessary endoscopic screening while maintaining patient safety.^20^

Regarding the variables linked to variceal presence, our stratification study showed some intriguing patterns. Males had the highest variceal prevalence, which is corresponding to the known gender discrepancy in portal hypertension and cirrhosis development, even though no clinical or demographic predictor attained statistical significance, most likely because of sample size limits.^21^ In contrast to NAFLD and autoimmune hepatitis, viral hepatitis etiology is associated with a greater variceal prevalence. This might be due to variations in fibrogenic pathways and disease development patterns. In contrast to metabolic etiologies, cirrhosis caused by viral hepatitis tends to acquire clinically relevant portal hypertension sooner in the course of the illness. Although not statistically significant, the trend toward a greater variceal predominance in overweight people relative to normal weight and obese people is worth considering. This result could reflect the intricate connection between variceal growth, portal pressure, and body composition. Rocky et al reported that APRI had limited diagnostic performance for identifying clinically significant portal hypertension with a sensitivity of 56% and specificity of 68%.^21^

The Youden-derived optimal cut-off values identified in our cohort (APRI >0.64 and PC/SD ≤1684) differed substantially from the traditionally recommended cut-offs (APRI >0.5 and PC/SD ≤884). This finding supports the growing evidence that diagnostic thresholds may vary according to disease etiology, ethnicity, and patient characteristics. Population-specific validation of non-invasive markers has been increasingly recommended before widespread clinical implementation.^20^

### Strengths

The use of upper gastrointestinal endoscopy as the reference standard, calculation of diagnostic indices with 95% confidence intervals, ROC analysis with DeLong comparison, multivariable logistic regression, and reporting of effect sizes rather than relying solely on p-values. These methodological perspectives augment the robustness and clinical applicability of our findings.

### Limitations

It was conducted at a single tertiary care center, which may limit the generalizability of the results. The cross-sectional design precludes assessment of temporal changes in non-invasive markers. In addition, red wale signs were not recorded separately, preventing the direct application of the complete Baveno VII definition of high-risk varices. Finally, the small number of patients in the “Other” etiology subgroup resulted in quasi-complete separation during logistic regression, and these estimates should therefore be interpreted cautiously.

Overall, this study demonstrates that both APRI and the PC/SD ratio are valuable non-invasive markers for predicting EVs in patients with compensated CLD. APRI appears more suitable as an initial screening tool because of its excellent sensitivity and negative predictive value, whereas the PC/SD ratio is more useful for confirming disease because of its outstanding specificity. The identification of cohort-specific optimal cut-off values suggests that local validation may further improve the diagnostic performance of these inexpensive and readily available non-invasive markers before routine clinical

## CONCLUSION

Both APRI and PC/SD ratio demonstrated acceptable diagnostic performance for predicting EVs in patients with compensated CLD. These simple, inexpensive, and readily available non-invasive markers may assist in the initial risk stratification of patients, particularly in resource-limited settings where access to endoscopic services is restricted. However, upper gastrointestinal endoscopy remains the reference standard for the diagnosis of EVs and these biomarkers should be considered complementary screening tools rather than replacements for endoscopic evaluation. Further multicenter prospective studies are needed to validate these findings in larger and more diverse patient populations.

### Authors’ contributions:

**NM:** Idea generation, literature search, data collection, manuscript writing, and is responsible for the integrity of study.

**IH:** Study concept, data analysis and interpretation, supervision and approval of final manuscript.

**MK:** Proposal writing, and critical review.

All authors have read and approved the final manuscript.
